# Prioritising gender, equity, and human rights in a GRADE-based framework to inform future research on self care for sexual and reproductive health and rights

**DOI:** 10.1136/bmjgh-2019-002128

**Published:** 2020-03-30

**Authors:** Nandi Siegfried, Manjulaa Narasimhan, Carmen H Logie, Rebekah Thomas, Laura Ferguson, Kevin Moody, Michelle Remme

**Affiliations:** 1 Independent Clinical Epidemiologist, Cape Town, South Africa; 2 Department of Sexual and Reproductive Health and Research, including UNDP-UNFPA-UNICEF-WHO-World Bank Special Programme of Research, Development and Research Training in Human Reproduction, World Health Organization, Geneva, Switzerland; 3 Factor-Inwentash Faculty of Social Work, University of Toronto, Toronto, Ontario, Canada; 4 Guideline Review Committee Secretariat (Science Division), World Health Organization, Geneva, Switzerland; 5 Institute on Inequalities in Global Health, Keck School of Medicine, University of Southern California, Los Angeles, California, USA; 6 Independent Consultant, Amsterdam, The Netherlands; 7 International Institute for Global Health, United Nations University, Kuala Lumpur, Malaysia

**Keywords:** epidemiology, study design, public health, infections, diseases, disorders, injuries

## Abstract

**Introduction:**

In January 2019, the WHO reviewed evidence to develop global recommendations on self-care interventions for sexual and reproductive health and rights (SRHR). Identification of research gaps is part of the WHO guidelines development process, but reliable methods to do so are currently lacking with gender, equity and human rights (GER) infrequently prioritised.

**Methods:**

We expanded a prior framework based on Grading of Evidence, Assessment, Development and Evaluation (GRADE) to include GER. The revised framework is applied systematically during the formulation of research questions and comprises: (1) assessment of the GRADE strength and quality rating of recommendations; (2) mandatory inclusion of research questions identified from a global stakeholder survey; and (3) selection of the GER standards and principles most relevant to the question through discussion and consensus. For each question, we articulated: (1) the most appropriate and robust study design; (2) an alternative pragmatic design if the ideal design was not feasible; and (3) the methodological challenges facing researchers through identifying potential biases.

**Results:**

We identified 39 research questions, 7 overarching research approaches and 13 discrete feasible study designs. Availability and accessibility were most frequently identified as the GER standards and principles to consider when planning studies, followed by privacy and confidentiality. Selection and detection bias were the primary methodological challenges across mixed methods, quantitative and qualitative studies. A lack of generalisability potentially limits the use of study results with non-participation in research potentially highest in more vulnerable populations.

**Conclusion:**

A framework based on GRADE that includes stakeholders’ values and identification of core GER standards and principles provides a practical, systematic approach to identifying research questions from a WHO guideline. Clear guidance for future studies will contribute to an anticipated ‘living guidelines’ approach within WHO. Foregrounding GER as a separate component of the framework is innovative but further elaboration to operationalise appropriate indicators for SRHR self-care interventions is required.

**Key questions**

**What is already known?**

- Reliable methods to identify research gaps from WHO guidelines are lacking with gender, equity and human rights (GER) infrequently prioritised when formulating future research questions.

**What are the new findings?**

- A prior Grading of Evidence, Assessment, Development and Evaluation (GRADE)-based framework was revised to include mandatory stakeholder input and selection of relevant GER standards and principles and successfully applied to a WHO guideline on self-care interventions for sexual and reproductive health and rights (SRHR).
- 39 research questions, 6 research approaches and 16 discrete study designs for SRHR self-care were identified and the key methodological issues outlined.
- Availability and accessibility were the GER standards and principles most frequently identified as relevant.

**What do the new findings imply?**

- Application of the GRADE-informed framework to future WHO guidelines development processes has potential to harmonise research question formulation and to ensure more consistent consideration of GER across the organisation.
- The dashboard outlining future SRHR studies is expected to prove useful to researchers.

## Introduction

The mandate of the WHO is to develop global clinical and public health guidance that informs country-level healthcare policies, guidelines, programmes and services. The systematic process of WHO guideline development includes the identification and documentation of research gaps.^1^ This is intended to focus research to inform and strengthen new and existing WHO recommendations. However, wide variation in the quality, robustness, presentation and dissemination of research priorities across sets of WHO guidelines has been noted.^2^ Currently within the WHO transformation process, systematic approaches to coherent research agenda formulation are being explored.

In 2018, the WHO Department of Reproductive Health and Research embarked on a process to: (1) develop evidence-based recommendations on self-care interventions for sexual and reproductive health and rights (SRHR) and (2) delineate research gaps in the field. A Guidelines Development Group (GDG) was established comprising experts and stakeholders active in SRHR and/or self-care interventions. The GDG was regionally and gender representative and included representation from youth, vulnerable populations, healthcare providers, policymakers, programme managers, researchers and civil society, as well as experts in human rights, gender equality and health economics.^3^

The following definition of self-care was adopted for the guidelines: *self-care is the ability of individuals, families and communities to promote health, prevent disease, maintain health and to cope with illness and disability with or without the support of a healthcare provider*.^4^ The scope of self-care as described in this definition includes health promotion, disease prevention and control, self-medication, providing care to dependent persons, seeking hospital/specialist care if necessary and rehabilitation including palliative care.

Members of the GDG attended a scoping meeting in March 2018 to prioritise and refine the self-care interventions to be evaluated in the guideline and to reflect on discussions arising from a prior WHO-led meeting on the ethical, legal, social accountability and human rights implications of self-care interventions.^5^ The GDG agreed that the systematic consideration of gender, equity and human rights (GER) in the provision of self-care interventions is key to ensuring better health for all. As defined by WHO, human rights-based approaches to health consider and address inequalities, power imbalances and discrimination, including those related to gender.^6^ Such approaches also aim to support better and more sustainable health and development outcomes and focus on capacity development, both of duty bearers to meet their obligations and of individuals to claim their rights.^7^ Attention to ways of empowering individuals and communities, particularly vulnerable populations, to understand and claim their rights requires special attention in many domains, including in research. Sridharan and colleagues^8^ argue that WHO needs to develop concrete actions towards mainstreaming GER. The GDG selected the fulfilment of the following GER standards and principles as key to measuring the impact of self-care interventions in future research studies: (1) the right to highest attainable standard of health (including availability, accessibility, acceptability and quality); (2) active and fully informed participation; (3) non-discrimination; (4) the right to seek, receive and impart information; (5) informed decision-making; (6) privacy and confidentiality; and (7) accountability.^9^

At a final GDG meeting in January 2019, the GDG reviewed evidence from five systematic reviews on self-care interventions in order to formulate new consensus-based self-care recommendations.^3^ The new recommendations covered the following topics: (1) self-injectable hormonal contraception, (2) over-the-counter oral contraception, (3) home-based ovulation predictor kits (OPKs), (4) self-sampling for human papilloma virus (HPV) and (5) self-collection of samples for sexually transmitted infections (STIs). Each recommendation was formulated in response to an a priori clinical or public health question regarding the effectiveness of the intervention when offered as an additional approach to current practice within the formal health sector. The questions were formulated using the Population, Interventions, Comparison and Outcomes (PICO) structure.^1^

To further inform the guidelines, the GDG also reviewed evidence from a global online survey of healthcare providers and users of healthcare services, which was hosted on the WHO website and shared via several listservs between July and October 2018.^10^ The survey included a range of questions regarding respondents’ values and preferences for self-care interventions for sexual and reproductive health. Three of these were interventions evaluated in the systematic reviews: (1) self-injectable hormonal contraception, (2) oral contraception (including over the counter), and (3) self-collection of samples for STIs (including HPV). The survey reached 294 (35.6%) healthcare providers and 531 (64.4%) users of healthcare services from 113 countries. There was diversity in WHO regional representation in responses from healthcare providers (Africa 30.0%, Europe 20.8%, Latin America and the Caribbean 20.8%, Asia 14.2%, Northern America 13.9% and Oceania 0.3%) and lay respondents (Europe 32.0%, Africa 23.0%, Asia 20.2%, Northern America 12.9%, Latin America and the Caribbean 10.5% and Oceania 1.3%).^10^

In this article, we present a systematic approach to the formulation of research questions to guide future studies related to the five topics of the new WHO recommendations on self-care interventions. We demonstrate how evidence from systematic reviews, results from a global survey and consideration of GER, together informed question formulation. Our primary aim is to demonstrate both the feasibility and utility of using a structured process combined with a GER lens to identify research gaps within the context of developing a WHO guideline. A secondary aim is to provide researchers with a dashboard of potential evidence-informed research questions and related feasible study designs—and associated methodological challenges and GER considerations—to ensure responsive future research in the rapidly evolving field of SRHR self-care.

## Methods

We adopted a similar methodological approach to research formulation used in two previously published WHO guidelines.^11 12^ Prior to the GDG meeting, a systematic review, including meta-analysis where appropriate, was conducted for each of the five selected PICO questions.^13–17^ The overall certainty of evidence was rated as *high, moderate, low* or *very low* according to the Grading of Evidence, Assessment, Development and Evaluation (GRADE) approach.^18^ The certainty of evidence is dependent on the risk of bias, precision, consistency, directness of the results and other considerations such as publication bias. During the meeting, the GDG formulated a recommendation in response to the following GRADE domains: certainty of the evidence, balance of benefits and harms, resource use implications, user values and preferences, acceptability among healthcare providers and key stakeholders, feasibility, equity and human rights (See table 1). Recommendations were then further categorised by the GDG as *strong* or *conditional*. In general, strong recommendations are made when the quality of evidence is high and the benefits of an intervention clearly outweigh the harms, whereas conditional recommendations recognise that the quality of the evidence is low or that specific country contextual factors may determine the uptake of a recommendation.^19^

**Table 1:** Key domains that require consideration when formulating WHO recommendations

Factor	How the factor influences the direction and strength of a recommendation
Quality of the evidence	The quality of the evidence across outcomes critical to decision making will inform the strength of the recommendation. The higher the quality of the evidence, the greater the likelihood of a strong recommendation.
Values and preferences	This describes the relative importance assigned to health outcomes by those affected by them; how such importance varies within and across populations; and whether this importance or variability is surrounded by uncertainty. The less uncertainty or variability there is about the values and preferences of people experiencing the critical or important outcomes, the greater the likelihood of a strong recommendation.
Balance of benefits versus harms	This requires an evaluation of the absolute effects of both benefits and harms (or downsides) of the intervention and their importance. The greater the net benefit or net harm associated with an intervention or exposure, the greater the likelihood of a strong recommendation in favour or against the intervention.
Resource implications	This pertains to how resource intense an intervention is, whether it is cost–effective and whether it offers any incremental benefit. The more advantageous or clearly disadvantageous the resource implications are, the greater the likelihood of a strong recommendation either for or against the intervention.
Priority	The problem’s priority is determined by its importance and frequency (ie, burden of disease, disease prevalence or baseline risk). The greater the importance of the problem, the greater the likelihood of a strong recommendation.
Equity and human rights	The greater the likelihood that the intervention will reduce inequities, improve equity or contribute to the realisation of one or several human rights as defined under the international legal framework, the greater the likelihood of a strong recommendation.
Acceptability	The greater the acceptability of an option to all or most stakeholders, the greater the likelihood of a strong recommendation.
Feasibility	The greater the feasibility of an option from the standpoint of all or most stakeholders, the greater the likelihood of a strong recommendation. Feasibility overlaps with values and preferences, resource considerations, existing infrastructures, equity, cultural norms, legal frameworks and many other considerations.

Reproduced from the WHO 2014.^1^

**Figure 1:**
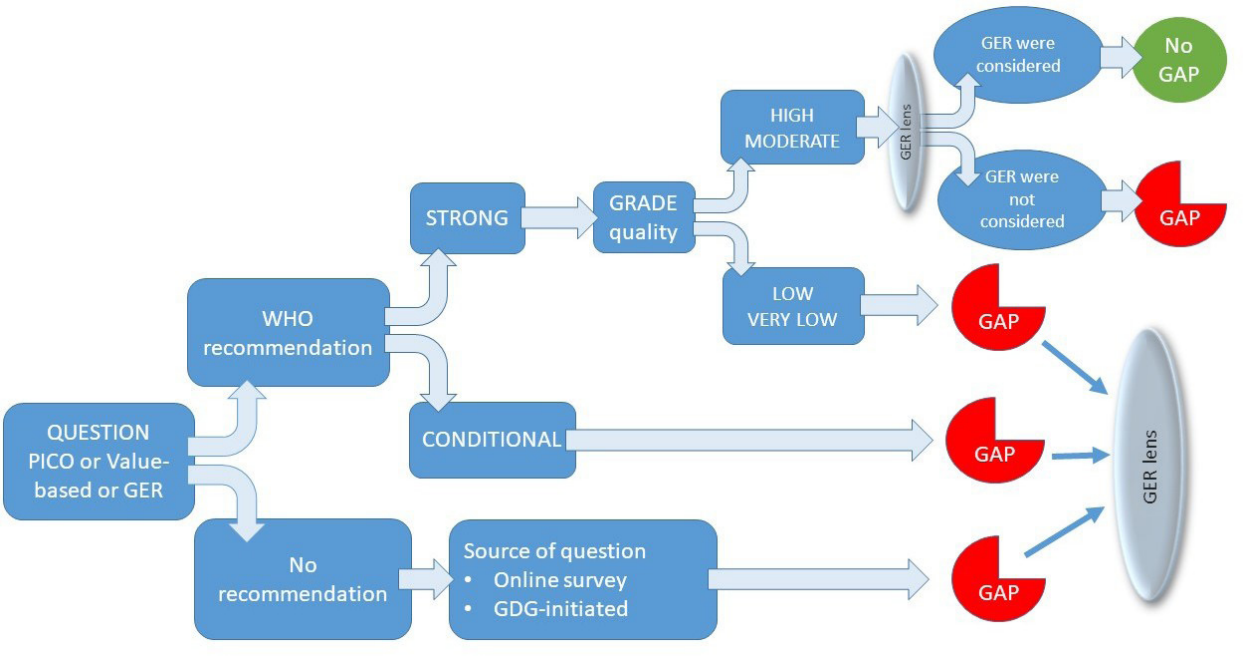
Hierarchical decision making algorithm to formulate research questions based on the presence and strength of a WHO recommendation, combined with the source of the question. GDG, Guidelines Development Group; GER, gender, equity and human rights; PICO, Population, Interventions, Comparison and Outcomes.

We used the GRADE framework as a starting point as it allows determination of research gaps based on the strength of the recommendation and the certainty of the evidence. For example, identification of a conditional recommendation, or low or very low certainty evidence, regardless of the strength of the recommendation, is indicative of where further research is required (see figure 1).

Previously, we had expanded the GRADE framework and included an additional component specific to the values and preferences of the community of users and potential users the recommendations intend to serve.^12^ We applied this step to the current guidelines on self-care interventions and reviewed results from the WHO-commissioned global online survey of users of sexual and reproductive self-care interventions.^3^ In addition, we captured specific research gaps identified by GDG participants during dedicated sessions at both the scoping and guidelines development meetings. We then viewed each research question through a GER lens and through discussion and consensus between authors of this paper identified the GER standards and principles essential to consider and measure when planning future research.

Following the GDG meeting, for each a priori PICO question, and for additional research questions identified from the global survey and during the meeting, we tabulated the following:

1. The clinical, programmatic, values-based or GER research question.
2. The related recommendation formulated by the GDG (or a record that no recommendation was made) where applicable.
3. The strength of each recommendation where applicable.
4. The certainty of the evidence underpinning the recommendation where applicable.
5. The GER standards and principles most relevant to the question as discussed during the scoping and guideline development meetings and agreed on by authors of this paper.

From the above, a research gap was identified when one or more of the following conditions was met: (1) the recommendation was conditional; or (2) the certainty of evidence was *low* or *very low* (even in the presence of a strong recommendation); or (3) key GER principles were not considered or absent (even if the certainty of evidence was *moderate* or *high* for clinical or public health effectiveness outcomes); or (4) no recommendation was made and the GDG or survey had articulated a research gap during their deliberations. We also identified a gap for strong recommendations if the evidence only arose from well-resourced settings. For each identified research gap, the authors then tabulated the following:

1. The most robust study design to answer the research question(s), including identification of GER principles to consider.
2. Conceptualisation of a pragmatic alternative study design if the ideal design was not feasible.
3. Consideration of the methodological challenges of the alternative study design with the potential bias(es) identified.

Following tabulation, we categorised the primary focus of each research question according to the relevant GRADE domain as outlined in table 1.

### Patient and public involvement

Participation of the public in developing research gaps was achieved in several ways: (1) through completion of the global survey, (2) members of several civil society associations contributed to the GDG meeting as participants on the GDG, (3) one of the authors is a patient representative and (4) all authors identify as individuals who engage or may engage with SRHR self-care interventions.

## Results

Tables 2–6 provide an overview of research gaps in SRHR self-care, the most relevant GER for each question and the study design(s) considered most feasible and appropriate to answer each question. Thirty-nine research questions were formulated with seven overarching research approaches and 13 discrete feasible study designs developed:

**Table 2:** Research gaps for self-administration of injectable contraception

Clinical, programmatic, values-based or human rights question		Current WHO recommendation	Strength of recommendation	Source or quality of evidence	Key GER considerations	Ideal study design(s)	Feasibility and practical constraints	Alternative study design	Methodological issues arising in the alternative study design
Benefits versus harms	PICO: should self-administered injectable contraception be made available as an additional approach to deliver injectable contraception?	Self-administered injectable contraception should be made available as an additional approach to deliver injectable contraception.	Strong.	Moderate.	No data available from LMIC; availability, accessibility, privacy and confidentiality.	RCT conducted in LMIC with inclusion of outcomes to measure accessibility, privacy and confidentiality.	As a strong WHO recommendation exists, it would be unethical to repeat an RCT of the intervention.	Prospective non-controlled cohort study to be conducted in several low- and middle-income countries where the intervention is or will be implemented. Analysis to combine quantitative survey supplemented with focused in-depth qualitative interviews with women (both those who have used and have not used self-administered injectable contraception and including adolescent girls and young women) to explore GER outcomes of availability, accessibility, privacy and confidentiality. Cost analyses can be nested in the cohort study.	*Confounding:* participants in cohort self-select to self-administer injectable and may have other similar characteristics that may influence their ability to access injectable contraception. *Measurement bias:* self-reporting bias an issue for qualitative component. This will require well trained researchers and validated tools. The triangulation of the qualitative data with the quantitative survey results provides an element of additional validation of outcomes.
Values and preferences	Is there an impact of differences between groups of women (eg, age, socioeconomic and occupational status) on their values and preferences with respect to self-administration of injectable contraception?	Not applicable.	Not applicable.	GDG.	Accessibility, privacy and confidentiality, non-discrimination.	Cross-sectional study of women using injectable contraception to evaluate associations between characteristics and use, or willingness to use.	A survey may not provide an objective measure or a true reflection of behaviours if driven by questions requiring socially acceptable responses.	This is a feasible study design but could be supplemented with diary collection of data as greater privacy and less likely for social desirability to influence self-recording. Partnering with women who have used or are willing to self-administer injectable contraception is important to identify critical and non-judgmental questions.	*Measurement bias:* for both survey and diary collection, questions will need to be formulated together with women using injectable contraception to ensure trust, participation and validity to avoid social desirability bias.
	What happens after women discontinue use of self-injectable contraception – do they use other methods?	Not applicable.	Not applicable.	GDG.	Accessibility.	National pharmacy or clinic-based registry of women using self-administered injectable contraception.	Registries can be costly to establish, maintain and monitor. A registry requires political will and capacity to provide effective data to inform decisions.	Convenience sample and survey of women attending family planning clinics to elucidate their prior modes of contraception and reasons for discontinuation.	*Selection bias*: such a sample would only include women who attend the clinic to change to a different form of contraception; women who cease to use self-administered contraception and do not return to the clinic would not be included. Their choices may differ from those women who do attend the clinic.
	What is the impact of stigma on the choice of self-administration of injectable contraception?	Not applicable.	Not applicable.	GDG and survey.	Accessibility, privacy and confidentiality and non-discrimination.	Qualitative study of interviews of women self-administering injectable contraception to elucidate their experiences and observations.	This is a feasible study.	Not applicable.	*Selection bias*: as with all qualitative studies, participants are purposively selected. However, this is an advantage in this study design as those women who have considered this issue will have rich experiences and observations to share.
	What are the optimal models of information provision for awareness raising and increasing knowledge of self-administration of injectable contraception?	Not applicable.	Not applicable.	GDG and survey.	Accessibility, the right to seek, receive and impart information.	Comparative effectiveness research of different models of information provision, optimally in an RCT.	This will first require development of models of dedicated information provision and health literacy (eg, telephonic, internet, posters and counselling) prior to experimental testing.	Exploratory qualitative interviews with women self-administering or willing to self-administer to identify their knowledge, attitudes and understanding of injectable contraception are required to inform models of information provision.	*Selection bias:* as with all qualitative studies, participants are purposively selected. However, this is an advantage in this study design as those women who have considered this issue will have rich experiences and observations to share which can guide the development of health promotion intervention and tools.
Human rights and equity	What implementation measures can ensure that inequity is reduced or minimised when self-administration is introduced?	Not applicable.	Not applicable.	GDG.	Non-discrimination.	Comparative effectiveness research of different models of implementation, optimally in a cluster RCT.	An RCT of models of implementation may be very costly due to the complex nature of implementation strategies that are dependent on the setting, type of provider and training required, and ensuring fidelity to the intervention.	Descriptive case studies of demonstration projects using different strategies to implement injectable contraception in programmes in several countries to indicate optimal measures to ensure equitable access and to formulate lessons learnt for scale-up elsewhere	*Lack of generalisability:* current practices and potential for scale will differ markedly between countries. Therefore selection of country sites for demonstration projects will require characterisation of underlying resource needs, training, staff and sociopolitical factors to ensure maximal diversity and that reasonable comparisons can be made between countries. Triangulation between data sources and sites will be necessary to increase generalisability.
Acceptability	Is there an impact of differences between healthcare providers (eg, age, income status of country, private/public sector) on the acceptability of self-administration of injectable contraception?	Not applicable.	Not applicable.	GDG.	Acceptability and accessibilty.	Cross-sectional study of healthcare providers to evaluate associations between characteristics and willingness to prescribe and provide injectable contraception for self-administration.	A survey may not provide an objective measure or a true reflection of behaviours if driven by questions requiring socially acceptable responses.	This is a feasible study design but could be supplemented with focus groups or qualitative interviews.	*Measurement bias:* for both survey and interviews, questions will need to be formulated together with healthcare providers to ensure trust, participation and validity and to avoid social desirability bias.
	What is the scale and consequence of incorrect use of self-administration?	Not applicable.	Not applicable.	GDG.	Accountability.	National pharmacy or clinic-based registry of women using self-administered injectable contraception.	Registries can be costly to establish, maintain and monitor. A registry requires political will and capacity to provide effective data to inform decisions.	Sentinel (active) surveillance through monitoring and evaluation of adverse effects and adverse events at clinic or provider level embedded in local and national quality assurance programmes.	*Reporting bias:* the data collected is dependent on the training and expertise of providers to adequately recognise adverse events. Resources can be directed towards appropriate sentinel sites to ensure staff are skilled and that data integrity is ensured.
Resource use	What are the user and provider costs of self-administration (as well as out-of-pocket expenditures), compared with provider-based administration?	Not applicable.	Not applicable.	GDG and survey.	Accessibility.	Empirical costing studies to estimate user and provider costs, and financing sources for each (users pay or health system pays) Data are currently only available from one study in three countries in sub-Saharan Africa. Given that costs vary by context and by delivery model, there is a need for more representative cost estimates to confirm that user costs and out-of-pocket expenditures are likely to decrease with self-administration.	This is a feasible study but can be costly to conduct across a representative sample of users and facilities.	This is feasible but could be supplemented by modelling based on the existing empirical cost data, cost structures and expert consultation on expected variation in parameters.	*Measurement bias*: the results are dependent on user recall and self-reported resource use, opportunity costs, as well as on assumptions regarding shared costs in health facilities.
	What is the cost-effectiveness of self-administration, compared with provider-based administration?	Not applicable.	Not applicable.	GDG.	Accessibility.	Modelling studies of cost- effectiveness across broader range of settings, taking both a healthcare provider and societal perspective.	This is a feasible study but its quality and usefulness depends on the reliability of the parameters for unit costs and effectiveness, which would be drawn from costing studies (noted above) and RCTs. It will also require more long-term modelling to capture downstream effects on the types of contraceptives self-injecting women switch to compared with provider-based injecting women.		*Measurement bias*: the results are dependent on selection of variables included in the model, assumptions regarding discount rates, cost-effectiveness thresholds and accurate estimates of current and projected costs and effects.
	What is the environmental impact of disposal of self-administered injectable oral contraception?	Not applicable.	Not applicable.	GDG.	Accountability.	Collection and collation of reports from environmental regulatory bodies documenting scale and impact of medical waste and household waste.	Regulatory agencies are unlikely to differentiate between whether injectable contraceptives present in waste are sourced directly from clinics or from the homes of users.	A qualitative household diary study of women who self-inject in which they record waste from the packaging and their management of it. This could be delivered with the injectable packaging and women can return it when collecting their next prescription.	*Recall bias:* women may not remember what they did with the packaging if they do not record it at the time. This may require an incentive during the research period in order to encourage women to complete the household waste diary.

GDG, Guideline Development Group; GER, gender, equity and human rights; RCTs, randomised controlled trials.

**Table 3:** Research gaps for self-collection of STI samples

Clinical, programmatic, values-based or human rights question		Current WHO recommendation	Strength of recommendation	Source or quality of evidence	Key GER considerations	Ideal study design(s)	Feasibility and practical constraints	Alternative study design	Methodological issues arising in the alternative study design
Benefits versus harms	PICO: should self-collection of samples for STIs be made available as an additional approach to deliver STI testing services?	Self-collection of samples for *Neisseria gonorrhoeae* and *Chlamydia trachomatis* should be made available as an additional approach to deliver STI testing services.	Strong.	Moderate.	No data from LMIC. Availability and accessibility.	RCT conducted in LMIC with inclusion of outcomes to measure availability and accessibility.	As a strong WHO recommendation exists, it would be unethical to repeat an RCT of the intervention.	Prospective non-controlled cohort study to be conducted in several LMICs where the intervention is or will be implemented. Analysis to combine quantitative survey supplemented with focused in-depth qualitative interviews with general population (with attention given to ensure sampling of vulnerable populations) to explore GER outcomes of availability and accessibility. Cost analyses can be nested in the cohort study.	*Confounding:* participants in cohort self-select to self-collect and may have other similar characteristics that may influence their ability to access STI self-collection. *Measurement bias:* self-reporting bias an issue for qualitative component. This will require well-trained researchers and validated tools. The triangulation of the qualitative data with the quantitative survey results provides an element of additional validation of outcomes.
		Self-collection of samples for *Treponema pallidum (syphilis*) and *Trichomonas vaginalis* may be considered as an additional approach to deliver STI testing services.	Conditional.	Low.	Availability, accessibility and acceptability.	Randomised controlled trial specific to self-collection of these organisms.	This is a feasible design.		*Generalisability*: the choice of setting and user population will determine how widely the results can be applied. The focus should be on testing this in LMIC settings and with vulnerable populations.
	What is the impact of self-sampling for STIs on partner screening?			GDG.	Privacy and confidentiality.	Randomised controlled trial with uptake of partner screening included as an outcome.	As a strong WHO recommendation exists, it would be unethical to repeat an RCT of the intervention.	Cross-sectional study of women who respond to results following STI self-collection to ascertain how many have notified their partners to be screened, and of those how many partners have gone for screening. Acceptability of screening for partners, and availability and accessibility of partner screening will be important to measure.	*Selection bias*: surveys will only capture those women who have responded actively to the results and may not be representative of those women who do not respond actively to their results.
	What is the impact of self-sampling for STIs on linkage to care and case-finding?			GDG.	Acceptability and non-discrimination.	RCT with inclusion of outcomes to measure linkage to care and case-finding.	As a strong WHO recommendation exists, it would be unethical to repeat an RCT of effectiveness of the intervention.	Interrupted time series using repeated cross-sectional surveys of populations attending STI clinics for treatment before, during and after implementation of policy to ascertain proportion of women who self-sampled prior to linkage to care.	*Attrition bias*: the results may indicate that more people who link to care used self-sampling, but this does not provide information on how many people who self-sampled with a positive result did not link to care.
	What is the benefit and harm of self-sampling for STIs of viral aetiology?			GDG.	Availability, accessibility and acceptability.	Randomised controlled trial specific to self-collection of STIs of viral aetiology. Outcomes to include measurement of availability and accessibility, as well as acceptability of self-sampling, especially regarding sampling of bloods (eg, hepatitis B virus).	This is a feasible design.		*Performance bias:* it is not possible to mask users or providers to mode of sampling. This can be mitigated by considering conducting a cluster RCT rather than an individually randomised trial, or a stepped wedge design where the intervention is delivered at staggered time periods.
Values and preferences	What are the values and preferences of marginalised populations (eg, Men who have sex with men, sex workers, trans populations) regarding self-sampling?	Not applicable.		GDG and survey.	Accessibility acceptability, privacy and confidentiality, and non-discrimination.	Qualitative key informant study of women and men who are marginalised regarding their values and preferences of self-sampling and the barriers that they experience or may experience when accessing self-sampling, receiving their results and when linking to care.	This is a feasible study.		*Selection bias*: as with all qualitative studies, participants are purposively selected. However, this is an advantage in this study design as marginalised women and men—including trans populations—who have considered this issue will have rich experiences and observations to share.
Human rights and equity	What are the best practices for avoiding coercion in self-sampling?	Not applicable.		GDG.	Non-discrimination, privacy and confidentiality.	Qualitative study using focus groups of vulnerable populations in contexts where self-sampling is implemented to understand how they perceive coercion could be avoided. This can allow creation of an expanded version of the intervention for further evaluation with measurement of non-coercion included as an outcome.	This is a feasible design.		*Selection bias*: as with all qualitative studies, participants are purposively selected. However, this is an advantage in this study design as marginalised women and men who have considered this issue will have rich experiences and observations to share.
Resource use	Is self-collection for STIs cost-effective?	Not applicable.	Not applicable.	GDG.	Accessibility.	Modelling studies of cost- effectiveness across broader range of settings, taking both a healthcare provider and societal perspective.	This is a feasible study, but its quality and usefulness depend on the reliability of the parameters for unit costs and effectiveness, which would be drawn from costing studies and the RCTs with effectiveness data. It will also require more long-term modelling to capture downstream effects on linkage to care.		*Measurement bias*: the results are dependent on selection of variables included in the model, assumptions regarding discount rates, cost-effectiveness thresholds and accurate estimates of current and projected costs. For example, vulnerable populations with limited access to care are likely to generate different results based on the probability that they would have limited or no access to care without self-sampling. *Data accuracy:* unit costs may not be available for many or any LMIC, especially where self-sampling has not been available. To avoid assumptions, this may require prior assessment via an initial implementation process to ascertain reasonable costings.
	Does self-collection offer a cost-efficient approach to case detection in low-income settings?	Not applicable.	Not applicable.	GDG.	Availability and accessibility.	Empirical costing studies to estimate the costs and targeting efficiency of self-collection in LMICs using various feasible models of delivery.	As above.		As above.

GDG, Guideline Development Group; GER, gender, equity and human rights; PICO, Population, Interventions, Comparison and Outcomes; RCTs, randomised controlled trials; STI, sexually transmitted infection.

**Table 4:** Research gaps for over-the-counter (OTC) oral contraception

Clinical, programmatic, values-based or human rights question		Current WHO recommendation	Strength of recommendation	Source or quality of evidence	Critical human rights and equity considerations	Ideal study design(s)	Feasibility and practical constraints	Alternative study design	Methodological issues arising in the alternative study design
Benefits versus harms	PICO: should OTC contraception be made available without a prescription?	OTC oral contraception should be made available without a prescription.	Strong.	Very low.	Privacy and confidentiality; accessibility.	RCT conducted in LMIC with inclusion of outcomes to measure accessibility, privacy and confidentiality.	As a strong WHO recommendation exists, it would be unethical to repeat an RCT of the effectiveness of the intervention.	Repeated cross-sectional surveys (can be targeted to specific subgroups or of women in the general population including adolescent girls and young women) attending pharmacies to evaluate privacy and confidentiality and choice to obtain OTC contraception over time.	*Selection bias*: women who participate in the survey may have fewer concerns regarding privacy and confidentiality than those who would refuse to participate, limiting generalisability.
	Are there quality differences between OTC oral contraception medication and prescribed oral contraception medication?	Not applicable.		GDG.	Not applicable.	This is a quality assessment concern and requires laboratory analysis and is outside the scope of this article. WHO guidelines for national regulatory authorities exist to guide national regulatory control to ensure substandard products are not made available and that dosages are correct (see https://www.who.int/medicines/areas/ quality_safety/quality_assurance/GoodRegulatory_ PracticesPublicConsult.pdf).			
	What are the optimal ways to provide advice on switching to different oral contraception or using other contraceptive options (eg, via text messaging, in person in pharmacy)?	Not applicable.		GDG.	Accessibility and informed decision making.	Comparative effectiveness research to compare different types of methods to provide advice.	A controlled trial that may or may not be randomised is feasible. Identification or development of modes of health literacy will be required prior to testing in a controlled manner. In LMIC, pharmacy technicians or cashiers may provide advice instead of pharmacists that requires consideration when planning real-world studies.		*Fidelity bias:* delivery of health advice is dependent on mode of advice. If given by pharmacists, community health workers or technicians, then monitoring the fidelity of the intervention will ensure the rigour of the results. Outcomes must include a measurement of accessibility such as requests for and uptake of advice.
	What adverse events arise from providing oral contraception OTC?	Not applicable.		GDG.	Acceptability, informed decision making and non-discrimination.	Convenience sample and cross-sectional survey of women obtaining OTC contraception at pharmacies to elucidate if they have experienced adverse events related to the OTC nature of the contraception.	This is a feasible study design.	Not applicable.	*Selection bias*: such a sample would only include women who are willing to provide information and who feel sufficiently confident to voice their concerns; women who have already experienced stigma or who fear it are less likely to return to the pharmacy and so are less likely to be represented in the sample.
Values and preferences	What are the values and preferences of women living in low-income and middle-income countries related to OTC oral contraception?	Not applicable.		GDG and survey.	Accessibility, privacy and confidentiality, and non-discrimination.	Qualitative study using key informant interviews with women seeking and/or using contraception and who live in LMIC regarding their values and preferences to elucidate their experiences and observations. A question regarding receipt of appropriate screening for contraindications will be essential to include.	This is a feasible study.	Not applicable.	*Selection bias*: as with all qualitative studies, participants are purposively selected. However, this is an advantage in this study design as those women who have considered this issue will have rich experiences and observations to share.
Human rights and equity	Will women of all ages be able to access OTC oral contraception? What barriers remain in the healthcare sector?	Not applicable.		GDG.	Availability, accessibility and non-discrimination.	Unannounced standard patient (simulation) study where women of different ages pose as users wishing to procure OTC contraception in different settings, regions and countries.	This is a feasible study design.	While this design is feasible, current obstacles to conducting standard patient research include automated electronic medical records and creation of believable identities. However, this should be less relevant for OTC studies than medical practice studies where simulation is widely used.	*Measurement error:* the presentation and responses of the 'standard user' may differ between scenarios and settings leading to overestimating or underestimating the accessibility of the OTC contraception. This can be addressed by ensuring adequate training for 'standard users' and developing templates for different responses.
Acceptability	What do healthcare providers know, think and feel about provision of OTC oral contraception, especially in low-income and middle-income settings?	Not applicable.		Survey.	Accessibility, privacy, the right to seek, receive and impart information.	Online global cross-sectional survey of healthcare providers with delivery through relevant organisations.	In many settings of deprivation, and especially in LMIC, online accessibility is limited by costs, inadequate technology and lack of knowledge on how to use tools.	Focus groups in purposively sampled healthcare provider populations and regions. Focus groups are most suitable to drive conversations and elicit responses among participants with shared lived experiences and is less confrontational as the aim is not to gather in-depth personal experiences but assess current cultural and social norms, practices and concerns with respect to OTC contraception. Specific questions can be directed regarding attitudes towards vulnerable populations, for example, trans-men and bisexual women.	*Social acceptability bias:* given that experiences are shared in a group, there may be a reluctance to disclose stigmatising behaviours; however, development of semistructured interview questions in consultation with local healthcare providers to best reflect local beliefs for interrogation can reduce this bias. In addition, a skilled focus group leader can create a safe space for disclosure.
Resource use	Who bears the cost of OTC oral contraception – is the cost shifted from the health system to the user?	Not applicable.		GDG.	Accessibility.	Regional patient costing study using cross-sectional survey of women obtaining OTC contraception at pharmacies to determine 'user cost pathway' (including transport; loss of income) and identify financing sources (eg, user pays, tax-based or contributory health insurance scheme pays).	This is a feasible study.		*Generalisability:* the results of any costing study will be specific to the context; however, the method of data collection can be replicated in other regions to determine locally specific costs. *Measurement bias:* much of the data will be dependent on participant recollections (recall bias may be a risk) and their own assumptions about what constitutes opportunity cost, for example.

GDG, Guideline Development Group; PICO, Population, Interventions, Comparison and Outcomes; RCT, randomised controlled trial.

**Table 5:** Research gaps for self-sampling for human papilloma virus (HPV) infection

Clinical, programmatic, values-based or human rights question		Current WHO recommendation	Strength of recommendation	Source or quality of evidence	Key GER considerations	Ideal study design(s)	Feasibility and practical constraints	Alternative study design	Main methodological issues arising in the alternative study design
Benefits versus harms	PICO: should HPV self-sampling be made available to adult women as an additional approach to clinician-based sampling and cervical services?	HPV self-sampling should be made available to adult women as an additional approach to clinician-based sampling and cervical services.	Strong.	Moderate.	No data from LMIC. Availability and accessibility.	RCT conducted in LMIC with inclusion of outcomes to measure availability and accessibility.	As a strong WHO recommendation exists, it would be unethical to repeat an RCT of the effectiveness of the intervention.	Prospective non-controlled cohort study to be conducted in several LMIC where the intervention is or will be implemented. Analysis to combine quantitative survey supplemented with focused in-depth qualitative interviews with women to explore GER outcomes of availability and accessibility. Cost analyses can be nested in the cohort study.	*Confounding:* participants in cohort self-select to self-sample and may have other similar characteristics that may influence their ability to access HPV self-sampling. *Measurement bias:* self-reporting bias an issue for qualitative component. This will require well trained researchers and validated tools. The triangulation of the qualitative data with the quantitative survey results provides an element of additional validation of outcomes.
Values and preferences	What is the optimal way(s) to engage women to self-sample, for example, via text or via community-based means?	Not applicable.		GDG.	Accessibility.	Comparative effectiveness research to compare different types of methods to engage women.	A controlled trial that may or may not be randomised is feasible. Identification or development of methods of engagement during a formative stage will be required prior to testing in a controlled manner. Current methods employed in HIV adherence programmes may be informative.		*Detection bias*: the outcome will be number of women engaged for each method. Ascertainment of engagement will likely be via self-report and as women will be aware of what method they received, there is a risk of detection bias. This can be reduced by ensuring that outcome assessors are masked to the allocated group, but a risk of detection bias remains.
Human rights and equity	What are the optimal methods to encourage women living in humanitarian settings to self-sample?				Availability, accessibility, acceptability, non-discrimination, privacy and confidentiality	Qualitative key informant study of users in humanitarian settings regarding their experiences of healthcare and desires for self-sampling.	This is a feasible study.		*Selection bias*: as with all qualitative studies, participants are purposively selected. However, this is an advantage in this study design as users and potential users living in humanitarian settings will have rich experiences and observations to share.
	How can linkage to care be ensured following HPV self-sampling?			GDG	Accessibility, acceptability and non-discrimination.	Comparative effectiveness research of a package of care that includes self-sampling combined with different strategies such as training of clinic staff to encourage women and facilitate linkage to care compared with self-sampling only.	A controlled trial that may or may not be randomised is a feasible design. Linkage to care needs to include links to care for women self-sampling and sensitisation to HPV vaccination and potential messaging to partners.		*Social desirability bias:* women will be aware of the different strategies they have received and may feel compelled to report positive experiences from staff, rather than discrimination. To address this, outcome assessment should be done by those blinded to the group and who are not part, or perceived to be part, of the clinic staff.
	What are the optimal methods to access homeless women?				Accessibility, acceptability and non-discrimination.	This is an under-researched area. Initial research should include identification of current health-seeking practices of homeless women via qualitative interviews with those working with the homeless and the homeless to find out how they would like to be reached and to develop strategies prior to further evaluation.	This is a feasible design.		*Lack of generalisability:* the results will be context specific and may not be generalisable to other regions; this is especially true of access to services. However, the desire for approaches to outreach may be generalisable to other settings.
Resource use	What is the cost-effectiveness of self-sampling when linkage to care is included as an outcome in the analysis?	Not applicable.	Not applicable.	GDG.	Availability and accessibility.	Modelling studies of cost- effectiveness across broader range of settings, taking both a healthcare provider and societal perspective.	This is a feasible study, but its quality and usefulness depend on the reliability of the parameters for unit costs and effectiveness, which would be drawn from costing studies and the RCTs effectiveness data. It will also require more long-term modelling to capture downstream effects on linkage to care.		*Measurement bias*: the results are dependent on selection of variables included in the model, assumptions regarding discount rates, cost-effectiveness thresholds and accurate estimates of current and projected costs. For example, vulnerable populations with limited access to care are likely to generate different results based on the probability that they would have limited or no access to care without self-sampling.
	What are the differences in out-of-pocket expenditures for self-sampling between high-income and low-income regions?	Not applicable.	Not applicable.	GDG.	Accessibility.	Empirical costing studies to estimate user and provider costs, and financing sources for each (users pay, or health system pays)	This is a feasible study.		*Measurement bias*: the results are dependent on user recall and self-reported resource use, opportunity costs, as well as on assumptions regarding shared costs in health facilities.

GDG, Guideline Development Group; GER, gender, equity and human rights; PICO, Population, Interventions, Comparison and Outcomes; RCT, randomised controlled trial.

**Table 6:** Research gaps for home-based ovulation predictor kits (OPKs)

Clinical, programmatic, values-based or human rights question		Current WHO recommendation	Strength of recommendation	Source or quality of evidence	Key GER considerations	Ideal study design(s)	Feasibility and practical constraints	Alternative study design	Main methodological issues arising in the alternative study design
Benefits versus harms	PICO: should home-based OPKs be made available as an additional approach to fertility management for women and couples desiring pregnancy?	Home-based OPKs should be made available as an additional approach to fertility management for women and couples desiring pregnancy.	Strong.	Very low.	No data from LMIC. Key GER considerations include availability and accessibility.	RCT conducted in LMIC with inclusion of outcomes to measure availability and accessibility, and exploration of potential for coercion.	As a strong WHO recommendation, it would be unethical to repeat an RCT of the effectiveness of the intervention.	Prospective non-controlled cohort study to be conducted in several LMIC where the intervention is or will be implemented. Analysis to combine quantitative survey supplemented with focused in-depth qualitative interviews with women and partners to explore GER outcomes of availability and accessibility. Exploration of potential for coercion would be advantageous to determine potential harms. Cost analyses can be nested in the cohort study.	*Confounding:* participants in cohort self-select to use OPK and may have other similar characteristics that may influence their ability to access OPKs. *Measurement bias:* self-reporting bias an issue for qualitative component as well as social desirability bias if both the women and partners are included. This will require well trained researchers and validated tools. The triangulation of the qualitative data with the quantitative survey results provides an element of additional validation of outcomes.
	How prevalent is infertility in low-income and middle-income settings and what are the consequences?	Not applicable.		GDG.	Not applicable.	Cross-sectional survey of women from general population in LMIC to ascertain self-reported prevalence of infertility. Cross-sectional semen analysis survey of selected young male populations in LMIC (eg, soldiers) to ascertain male infertility.	A large general population survey is feasible but has cost implications to achieve a sufficiently large sample to be representative.	Focused cross-sectional study of women attending primary care clinics, excluding antenatal care, to ascertain self-reported prevalence of infertility.	*Berkson’s bias:* people who present with one condition are more likely to have a second condition. Therefore, sampling from a clinic may over-represent the prevalence of infertility and not be representative of the general population.
	What is the impact of using a home-based OPK on communication between partners?	Not applicable.		GDG.	Privacy and confidentiality.	Pharmacy-led survey of women's experiences of partner communication when purchasing the OPK and after using the OPK.	Privacy and perception of lack of privacy, may compromise the integrity of the data and women's willingness to consent to participate. Lack of trained professionals in the pharmacy may also reduce the uptake and quality of the survey.	Qualitative key informant interviews of couples invited to participate either at infertility clinics or at pharmacies.	*Social acceptability bias:* this study will require careful interviewing with highly trained interviewers to elicit responses that are truthful and not socially desirable. Interviewing women and men separately may reduce this but consequently reduces the observation of communication patterns between partners.
Values and preferences	What are the values and preferences of women and men regarding infertility in settings of deprivation (both in high-income countries and in low-income and middle-income countries)?	Not applicable.		GDG.	Non-discrimination.	Online global cross-sectional survey of men and women delivered through relevant organisations. The participants can be any age and infertility is not an inclusion criteria.	In many settings of deprivation, and especially in LMIC, online accessibility is limited by costs, inadequate technology and lack of knowledge on how to use tools. Specific groups, for example, trans men, may not be reached by a survey aimed at the general population.	Focus groups in purposively sampled populations and regions. Focus groups are most suitable to drive conversations and elicit responses among participants with shared lived experiences and is less confrontational as the aim is not to gather in-depth personal experiences of infertility, but assess current cultural and social norms. Recruitment of specific groups, for example, trans men, can be targeted appropriately.	*Social acceptability bias:* given that experiences are shared in a group, there may be a reluctance to disclose stigmatising behaviours; however, development of semistructured interview questions in consultation with researchers and representatives of the sampled population to best reflect local beliefs for interrogation can reduce this bias. In addition, a skilled focus group leader can create a safe space for disclosure.
Resource use	Are home-based OPKs cost-effective compared with other fertility management options?	Not applicable.		GDG.	Availability and accessibility.	Modelling studies of cost-effectiveness.	This is a feasible study design. Modelling will require identification of secondary data on the costs of OPK and other fertility management options in a specific country setting. Assumptions underlying the model will include selection of a primary outcome (pregnancy or live birth) or disability-adjusted life years or quality-adjusted life years (which can capture reduced disability or improved quality of life from reduction in anxiety). Assessment and incorporation of human rights measurements such as privacy and non-coercion will be challenging. Any modelling will either need to be country-specific or stratified by country.		*Lack of generalisability*: a cost comparison would provide indirect data for many regions where differences between costs of fertility management options and costs of access to healthcare differ widely.

GDG, Guideline Development Group; GER, gender, equity and human rights; PICO, Population, Interventions, Comparison and Outcomes; RCT, randomised controlled trial.

1. Survey approach (one prevalence survey; two interrupted time series; five cross-sectional studies; one household diary study).
2. Qualitative approach (seven key informant interviews; three focus groups).
3. Implementation research approach (four prospective mixed methods cohort studies; one demonstration project).
4. Comparative effectiveness research (three non-randomised controlled trials (non-RCTs); two RCTs).
5. Economic approach (four cost-effectiveness studies; four costing studies).
6. Surveillance (one sentinel surveillance study).
7. Standard patient study (1).

We identified selection bias and detection bias as the primary methodological challenges across mixed methods, quantitative and qualitative studies. Detection bias was driven by concerns around self-reported data that may be prone to social desirability bias when participants provide answers they consider the assessors expect to hear. Selection bias in non-randomised studies limits generalisability and occurs when research participants who agree to participate differ qualitatively from those who do not agree to participate. Non-participation may be highest in those vulnerable populations who fear discrimination or have privacy and confidentiality concerns. This would then limit the utility of the results obtained from those who do participate, including in relation to GER.

The most frequent GER standards and principles considered relevant were availability and accessibility, followed by privacy and confidentiality.

## Discussion

We developed a structured approach to identification of research gaps and formulation of research questions and study designs during a WHO guidelines development process. Our approach builds on our previous work in this area and is rooted in the current GRADE framework used by WHO, further extending it to incorporate the user perspective and foregrounding GER throughout the process.

To the best of our knowledge, our approach is unique in providing practical systematised steps to research question formulation during WHO guideline development and elaboration of the design of future studies. Prior work in this area has been sparse and focused on identification and characterisation of research gaps arising from systematic reviews (not guidelines) and has not described the type of research that is required.^20^ Given that the aim of identification of research gaps is to reduce waste and increase research value,^21^ collation and possible registration of research gaps on a publicly accessible platform should be encouraged to realign future studies with the existing body of evidence. This would increase access to suggested methodologies and processes to advance research from resource-constrained settings where we found gaps in existing evidence around self-care interventions. Given that much of the evidence reviewed came from high-income countries, it will also be important for future research in these fields to develop capacity where needed and promote research appropriate to the local contexts in low-income and middle-income countries.

The application of a ‘living guidelines’ approach to WHO guideline development process will ensure greater responsiveness to new research findings.^22^ Current guidance for formulating and prioritising research gaps during WHO guideline development processes requires strengthening in the *WHO Handbook of Guideline Development*.^1^ Research gap identification is rarely prioritised during guideline development processes and the opportunity to shape the future research agenda is missed.^2^ Our practical approach presents an opportunity to WHO (and other guidelines developers) to better integrate research identification and elaboration into the guidelines decision-making space, in particular research that is reflective of need and priorities. Time and cost constraints may preclude extensive discussion of research gaps during a guidelines meeting, but at a minimum a dedicated agenda item and a working group tasked to develop the research gaps further following the meeting should be considered. We can envision automation of the decision-making steps in our approach using the hierarchical model outlined in figure 1. However, we would caution that such a process be viewed as a starting point to facilitate more in-depth discussions rather than a rigid template.

GER are recognised as key considerations in the SRHR field.^23 24^ SRHR self-care interventions present many opportunities to address common obstacles to delivering equitable, gender-responsive and rights-based healthcare. Provided users are fully informed, able to make autonomous choices and are able to link to a health system when required, high-quality self-care interventions can offer available, accessible and acceptable healthcare to those individuals and groups who may be less likely to access formal healthcare due to fear of discrimination or privacy and confidentiality concerns. Benefits may flow to healthcare providers too as users become more engaged in their healthcare, with task-shifting reducing heavy workloads and consumer self-sampling potentially reducing the risk of personal injury to providers when obtaining samples (eg, via venepuncture).

We consistently identified *accessibility* as an important GER principle to measure across questions for all five interventions. From a human rights perspective, *accessibility* encompasses physical accessibility, economic accessibility (affordability), non-discrimination in access and information accessibility.^25^ This illustrates that multiple indicators are often required to measure a single outcome domain. Within a rights-based approach to measuring health, health indicators can be used to measure different standards and principles relating to GER.^26^ For example, some health surveys collect data on informed choice in contraception uptake (measured by the proportion of users who were informed of potential side effects, what to do in the case of side effects and alternative contraceptive options). This highlights the human rights standard of acceptability as well as the principle of autonomy, expressed through free, full and informed decision making.^27^ Similarly, indicators commonly used in the fields of GER may be used to measure certain aspects of sexual and reproductive health; for example, measures of the existence, content and degree of implementation of relevant laws and policies governing access to sexual and reproductive health information and services can help elucidate patterns in uptake of services, including for self-care initiatives. An indicator that incorporates both sets of concern will allow for identification of interventions that are sensitive to issues relating to GER and most effective in terms of improving health.^26^

In our approach, we identified the GER standards and principles most relevant to each question but did not operationalise measurement. This is an important next step. Development of, and agreement on, standardised indicators and the optimal instruments to measure these within the SRHR self-care intervention field will greatly enhance the usability and uptake of GER-informed evidence into future guidelines through streamlining meta-analysis and synthesis more broadly. The experience and guidance of the Core Outcome Measures in Effectiveness Trials (COMET) initiative are instructive in this regard. COMET advocates for the development and application of agreed, standardised sets of outcomes, known as ‘core outcome sets’, which represent the minimum that should be measured and reported in all clinical trials of a specific condition and are also suitable for use in clinical auditing or research other than randomised trials.^28^ Inclusion of GER in an SRHR self-care core outcome set will ensure GER are integral to all future evaluations. We welcome initiatives to bring researchers from the human rights, gender, health economics and epidemiology fields together to advance this.

The WHO aims to be representative when determining GDG composition, and for the guideline on self-care interventions, we believe reasonable representation was achieved. However, in general, the balance is in favour of healthcare providers rather than community members and users of interventions. We were also able to ensure participation of users and healthcare providers of SRHR self-care interventions by incorporating the findings of the global survey and focus group discussions with vulnerable populations into the formulation of research gaps. Active and informed participation of users is also an important consideration during implementation of recommendations and in planning future research studies. Many of the proposed future studies are focused on vulnerable populations, and participation in the study or assessment of an outcome may place participants at risk. This illustrates that outcome measurement and the process of research itself requires careful consideration of GER to minimise unintended harms. Community-based participatory research is a useful strategy to conducting research that is relevant, appropriate and acceptable.

Prior to conducting studies on self-care, researchers need to consider the burdens faced by study participants, particularly for implementation research and qualitative methodologies. There is an opportunity cost to participants who may have to take time away from work or family care in order to contribute to research activities. We encourage meaningful involvement of individuals or groups representing target populations in the design and cocreation of research methodologies to optimise benefits to individuals and the wider group while minimising burdens to the participants. This approach is supported by ethical considerations^29^ and several frameworks that promote person-centred interventions.^30 31^

Each of the proposed study designs can be further expanded into a template for a study protocol and made publicly available. The WHO successfully achieved rapid deployment of ethically approved clinical trial protocols for the Ebola vaccine under emergency conditions.^32 33^ Under more controlled conditions, WHO may consider systematically developing field-specific study protocol templates that meet ethics standards. Researchers can then modify these templates to their context addressing any specific local ethics requirements before rapidly conducting studies during implementation of new WHO recommendations to inform programme scale-up or for testing new strategies well in advance of planned future GDG meetings.

Similar to our previous work where we applied the original framework to SRHR guidelines for women living with HIV, we found that many of the identified research questions require evaluation of complex, multifaceted and often multisectoral interventions that may be best suited to evaluation within an implementation research paradigm.^12^ Implementation science provides a platform to learn whether an intervention works in real-world settings and demonstrates how to ensure an intervention can effectively be brought to scale. Importantly, outcomes such as acceptability, feasibility and costs are encouraged in addition to conventional measures of effectiveness.^34^ RCTs are the optimal design for providing evidence of efficacy and will remain the gold standard for informing WHO recommendations. However, comparative effectiveness research (CER) where available interventions are compared with each other (instead of with usual care or placebo) can be viewed as a bridge between RCTs and implementation research.^35 36^ Five of our research questions are best evaluated using CER (eg, comparison on OPKs with other fertility management options) with 10 studies requiring a multistudy approach best articulated in implementation science. The challenge for WHO will be how these types of study designs and approaches can best be incorporated into future WHO guidelines decision making. Some progress has been made with the inclusion of the Risk of Bias in Non-randomised studies of interventions (ROBINS-I) tool for assessing risk of bias in non-randomised studies in GRADE,^36^ guidance for rating certainty of evidence when reviewing public health interventions^37^ and the development of the WHO-INTEGRATE framework to WHO guidelines development that includes specific methods to incorporate norms and values and a greater complexity perspective.^38^

Our approach is unique to the development of normative guidance. As such it is limited by the scope of a guideline, which may be intentionally narrow due to feasibility, resources and time constraints. The research gaps arising from such a process are specific to the scope of the guidelines and should not be viewed as a comprehensive research agenda for the relevant clinical or public health field nor as a research prioritisation exercise. However, the resultant research questions can provide a baseline list of questions that can then be further subjected to one of several research prioritisation methodologies.^39–41^

Lastly, while our overall decision-making process is structured and systematised, the selection of the most relevant GER standards and principles was done primarily through iterative discussion and ultimately consensus between study authors. Such decisions may not readily lend themselves to standardised processes, but we would advocate that in order to ensure the integrity and generalisability of the selected standards and principles, decisions should be made by a diverse and representative group as possible. We attempted to do so by reflecting a diversity of backgrounds, skills and experiences among the study authors, but we acknowledge that our selections are nonetheless subjective and may be best done by the broader GDG. Further exploration and development of ways to standardise such decisions such as initiated in the family planning field is now required.^42^

## Conclusion

A framework based on GRADE that includes stakeholders’ values and identification of core GER standards and principles provides a practical, systematic approach to identifying research questions from a WHO guideline. Uptake of this framework has the potential to harmonise methods and ensure more consistent consideration of research question formulation across the organisation. Clear guidance for future studies, including anticipation of, and methods to reduce risks of bias, can contribute to an anticipated ‘living guidelines’ approach within WHO. Foregrounding GER as a separate component of the framework is key to ensuring it is considered as integral to outcome evaluation, and further elaboration to operationalise appropriate indicators for SRHR self-care interventions is required.

## References

[1] World Health Organization WHO Handbook for Guideline development. 2nd edn, 2014 Available: https://www.who.int/publications/guidelines/guidelines_review_committee/en/ [Accessed 19 Mar 2020]

[2] Maher D, Ford N A public health research agenda informed by guidelines in development. Bull World Health Organ 2017;95:795-A 10.2471/BLT.17.200709 29200517 PMC5710088

[3] World Health Organization WHO consolidated guideline on self-care interventions for health: sexual and reproductive health and rights. Geneva: World Health Organization, 2019 Licence: CC BY-NC-SA 3.0 IGO 31334932

[4] Hatch S IK Self-help and health in Europe: new approaches in health care. Albany, NY: World Health Organization Publications Center USA, 1983.

[5] World Health Organization WHO meeting on ethical, legal, human rights and social accountability implications of self-care interventions for sexual and reproductive health: 12–14 March 2018, Brocher Foundation, Hermance, Switzerland: summary report. Geneva: World Health Organization, 2018 Licence: CC BY-NC-SA 3.0 IGO.

[6] World Health Organization Gender, equity and human rights, 2019 Available: https://www.who.int/gender-equity-rights/understanding/human-rights-definition/en/ [Accessed 5 Jun 2019].

[7] World Health Organization A human-rights based approach to health. Available: https://www.who.int/hhr/news/hrba_to_health2.pdf [Accessed 22 Aug 2019].

[8] Sridharan S, Maplazi J, Shirodkar A, et al Incorporating gender, equity, and human rights into the action planning process: moving from rhetoric to action. Glob Health Action 2016;9:30870 10.3402/gha.v9.30870 27606968 PMC5015636

[9] Ferguson L, Fried S, Matsaseng T, et al Human rights and legal dimensions of self care interventions for sexual and reproductive health. BMJ 2019;365:l1941 10.1136/bmj.l1941 31085551 PMC6511940

[10] World Health Organization WHO consolidated guideline on self-care interventions for health: sexual and reproductive health and rights web supplement: global values and preferences survey report. Geneva: World Health Organization, 2019 https://apps.who.int/iris/bitstream/handle/10665/329989/WHO-RHR-19.24-eng.pdf?ua=1

[11] Siegfried N, Beanland RL, Ford N, et al Formulating the future research agenda for postexposure prophylaxis for HIV: methodological challenges and potential approaches. Clin Infect Dis 2015;60(Suppl 3):S205–11. 10.1093/cid/civ139 25972506

[12] Siegfried N, Narasimhan M, Kennedy CE, et al Using GRADE as a framework to guide research on the sexual and reproductive health and rights (SRHR) of women living with HIV - methodological opportunities and challenges. AIDS Care 2017;29:1088–93. 10.1080/09540121.2017.1317711 28449598

[13] Yeh PT, Kennedy CE, Van der Poel S, et al Should home-based ovulation predictor kits be offered as an additional approach for fertility management for women and couples desiring pregnancy? A systematic review and meta-analysis. BMJ Glob Health 2019;4:e001403 10.1136/bmjgh-2019-001403 PMC6509595 31139458

[14] Yeh PT, Kennedy CE, de Vuyst H, et al Self-sampling for human papillomavirus (HPV) testing: a systematic review and meta-analysis. BMJ Glob Health 2019;4:e001351 10.1136/bmjgh-2018-001351 PMC6529022 31179035

[15] Ogale Y, Yeh PT, Kennedy CE, et al Self-collection of samples as an additional approach to deliver testing services for sexually transmitted infections: a systematic review and meta-analysis. BMJ Glob Health 2019;4:e001349 10.1136/bmjgh-2018-001349 PMC6509609 31139454

[16] Kennedy CE, Yeh PT, Gonsalves L, et al Should oral contraceptive pills be available without a prescription? A systematic review of over-the-counter and pharmacy access availability. BMJ Glob Health 2019;4:e001402 10.1136/bmjgh-2019-001402 PMC6606062 31321085

[17] Kennedy CE, Yeh PT, Gaffield ML, et al Self-administration of injectable contraception: a systematic review and meta-analysis. BMJ Glob Health 2019;4:e001350 10.1136/bmjgh-2018-001350 PMC6528768 31179026

[18] Guyatt GH, Oxman AD, Vist GE, et al GRADE: an emerging consensus on rating quality of evidence and strength of recommendations. BMJ 2008;336:924–6. 10.1136/bmj.39489.470347.AD 18436948 PMC2335261

[19] Andrews JC, Schünemann HJ, Oxman AD, et al Grade guidelines: 15. going from evidence to recommendation-determinants of a recommendation's direction and strength. J Clin Epidemiol 2013;66:726–35. 10.1016/j.jclinepi.2013.02.003 23570745

[20] Robinson KA, Saldanha IJ, McKoy NA Frameworks for determining research gaps during systematic reviews : Methods future research needs report No. 2. Rockville MD: Agency for Healthcare Research and Quality, 2011 www.effectivehealthcare.ahrq.gov/reports/final.cfm 21977524

[21] Dechartres A, Ravaud P Better prioritization to increase research value and decrease waste. BMC Med 2015;13:244 10.1186/s12916-015-0492-3 26416788 PMC4587677

[22] Akl EA, Meerpohl JJ, Elliott J, et al Living systematic reviews: 4. living guideline recommendations. J Clin Epidemiol 2017;91:47–53. 10.1016/j.jclinepi.2017.08.009 28911999

[23] World Health Organization Ensuring human rights in the provision of contraceptive information and services: guidance and recommendations, 2019 Available: https://apps.who.int/iris/bitstream/handle/10665/102539/9789241506748_eng.pdf;jsessionid=36DDE4C1852D56BB855F5082C1436DA7?sequence=1 [Accessed 30 Sep 2019]. 24696891

[24] World Health Organization Reproductive, maternal, newborn and child health and human rights: a toolbox for examining laws, regulations and policies, 2016 Available: https://apps.who.int/iris/bitstream/handle/10665/126383/9789241507424_eng.pdf;jsessionid=7A1BFE94AB8933086237F6D385966381?sequence=1 [Accessed 30 Sep 2019].

[25] UN Committee on Economic, Social and Cultural Rights (CESCR) General Comment No. 14: the right to the highest attainable standard of health (art. 12 of the covenant), 11 August 2000, E/C.12/2000/4. Available: https://www.refworld.org/docid/4538838d0.html [Accessed 10 Sep 2019].

[26] Gruskin S, Ferguson L Using indicators to determine the contribution of human rights to public health efforts. Bull World Health Organ 2009;87:714–9. 10.2471/BLT.08.058321 19784452 PMC2739915

[27] World Health Organization Ensuring human rights within contraceptive programmes: a human rights analysis of existing quantitative indicators, 2014 Available: https://apps.who.int/iris/bitstream/handle/10665/126799/9789241507493_eng.pdf?ua=1&ua=1?sequence=1 [Accessed 8 Oct 2019].

[28] Williamson PR, Altman DG, Bagley H, et al The COMET Handbook: version 1.0. Trials 2017;18:280 10.1186/s13063-017-1978-4 28681707 PMC5499094

[29] Aluwihare-Samaranayake D Ethics in qualitative research: a view of the participants' and researchers' world from a critical standpoint. Int J Qual Methods 2012;11:64–81. 10.1177/160940691201100208

[30] Narasimhan M, Allotey P, Hardon A Self care interventions to advance health and wellbeing: a conceptual framework to inform normative guidance. BMJ 2019;365:l688 10.1136/bmj.l688 30936087 PMC6441866

[31] World Health Organization Who framework on integrated person-centred services. Sixty-ninth World health assembly A69/39, 2016 Available: https://www.who.int/servicedeliverysafety/areas/people-centred-care/Overview_IPCHS_final.pdf?ua=1 [Accessed 20 Sep 2019].

[32] Calain P The Ebola clinical trials: a precedent for research ethics in disasters. J Med Ethics 2018;44:3–8. 10.1136/medethics-2016-103474 27573153 PMC5749307

[33] Heymann DL, Rodier GR, Ryan MJ Ebola vaccines: keep the clinical trial protocols on the shelf and ready to roll out. Lancet 2015;385:1913–5. 10.1016/S0140-6736(15)60645-6 25843891 PMC7133714

[34] Peters DH, Adam T, Alonge O, et al Implementation research: what it is and how to do it. BMJ 2013;347:f6753 10.1136/bmj.f6753 24259324

[35] Glasgow RE, Rabin BA Implementation science and comparative effectiveness research: a partnership capable of improving population health. J Comp Eff Res 2014;3:237–40. 10.2217/cer.14.9 24969150

[36] Schünemann HJ, Cuello C, Akl EA, et al GRADE guidelines: 18. How ROBINS-I and other tools to assess risk of bias in nonrandomized studies should be used to rate the certainty of a body of evidence. J Clin Epidemiol 2019;111:105–14. 10.1016/j.jclinepi.2018.01.012 29432858 PMC6692166

[37] Montgomery P, Movsisyan A, Grant SP, et al Considerations of complexity in rating certainty of evidence in systematic reviews: a primer on using the grade approach in global health. BMJ Glob Health 2019;4:e000848 10.1136/bmjgh-2018-000848 PMC6350753 30775013

[38] Rehfuess EA, Stratil JM, Scheel IB, et al The WHO-INTEGRATE evidence to decision framework version 1.0: integrating who norms and values and a complexity perspective. BMJ Glob Health 2019;4:e000844 10.1136/bmjgh-2018-000844 PMC6350705 30775012

[39] Nasser M, Welch V Prioritization of systematic reviews leads prioritization of research gaps and needs. J Clin Epidemiol 2013;66:522–3. 10.1016/j.jclinepi.2012.09.007 23265604

[40] Nasser M, Ueffing E, Welch V, et al An equity lens can ensure an equity-oriented approach to agenda setting and priority setting of Cochrane reviews. J Clin Epidemiol 2013;66:511–21. 10.1016/j.jclinepi.2012.11.013 23477991

[41] Bhaumik S, Rana S, Karimkhani C, et al Ethics and equity in research priority-setting: stakeholder engagement and the needs of disadvantaged groups. Indian J Med Ethics 2015;12:110–3. 10.20529/IJME.2015.030 25797432

[42] Gruskin S, Ferguson L, Kumar S, et al A novel methodology for strengthening human rights based monitoring in public health: family planning indicators as an illustrative example. PLoS One 2017;12:e0186330 10.1371/journal.pone.0186330 29220365 PMC5722344

